# Effect of WeChat consultation group on residents staying at home in Sichuan and Chongqing regions during the Coronavirus Disease 2019 (COVID-19) outbreak in China: a cross-sectional study

**DOI:** 10.1186/s12889-020-09951-4

**Published:** 2020-11-30

**Authors:** Xiaolei Hu, Xuejun Li, Yang Lü, Jing Tang, Hai Rong Li, Min Tang

**Affiliations:** 1 Department of Pharmacy, Southwest Hospital, Third Military Medical University (Army Medical University), Chongqing, China; 2 Hua Tai KuiGe Hospital of GuangAn, Guang’an, Sichuan China; 3 Department of Geriatrics, The First Affiliated Hospital of Chongqing Medical University, Chongqing, China; 4 Chongqing Niwen Education, Chongqing, China; 5 Chongqing HuiyiLogigistics Limited Company, Chongqing, China

**Keywords:** Coronavirus disease 2019, COVID-19, WeChat, Consultation, Disease

## Abstract

**Background:**

After the outbreak of Coronavirus in Wuhan, Hubei, in 2019, the disease rapidly spread to other parts of China as well as outside of China. Since the pandemic outbreak, the general public has been responsive to the national call to stay at home in quarantine. However, since doubts and anxiety related to the disease have been detected in the general public, in this study, we established the WeChat platform “Coronavirus Disease 2019 Voluntary Assistance Group in Sichuan and Chongqing regions” in January 2020, which was committed to providing professional consultation and psychological counseling services for residents in Sichuan and Chongqing during the Coronavirus Disease 2019 outbreak. Our aim was to analyze the consultation practices of residents in the WeChat assistance group and provide a reference for the similar “non-contact” voluntary service platforms aiming to implement consultations during the late pandemic period.

**Methods:**

A cross-sectional study was conducted using the records containing the consultation content from the WeChat assistance groups in Sichuan and Chongqing between January 30 and March 1, 2020. Data on consultation content, changes in a number of consultation items, answers, knowledge on popular science, and expert advice were summarized, and the Pareto chart was used to analyze the primary and secondary factors of consultation content.

**Results:**

The constituent ratio of “respiratory symptoms, masks, and disinfection” in consultation content ranked as the top three factors. Cumulatively, they occupied 49.77% of the content, thus resulting as the primary factors in the consultation content. The number of consultation items suddenly increased from 10 on the first day to 116 on the 7th day, resulting in a 1060% increased rate. There were 151 consultation factors, among which 130 (86.1%) were related to the Coronavirus Disease 2019, and 21 (13.9%) were unrelated to the Coronavirus Disease 2019.

**Conclusion:**

WeChat groups may be used as an effective means for providing assistance services for the public during the Coronavirus Disease 2019 outbreak.

## Background

In December 2019, Coronavirus Disease 2019 (COVID-19) that was first identified in Wuhan, Hubei, rapidly spread to other parts of China as well as outside of China. The outbreak coincided with China’s Spring Festival holiday when a large number of people are on the move, which further accelerated pandemic and led to a rapid increase in the number of confirmed cases. On January 31, 2020, the World Health Organization pointed out that the rapid spread of COVID-19 had become a global emergency and was declared as such [1].

Thus far, COVID-19 has been confirmed in 211 countries worldwide. The US Johns Hopkins University released data showing that: by April 11, 2020, the cumulative number of confirmed COVID-19 cases exceeded 1.7 million, reaching 1,715,143 cases, while the cumulative number of deaths reached 103,874 cases. The countries with the highest number of cumulative confirmed cases were the United States, Spain, Italy, France, and Germany. According to data released by the National Health Commission of the People’s Republic of China: by April 12, 2020, 83,482 confirmed cases and 3349 deaths have been reported nationwide. However, through concerted efforts across the country and joint anti-pandemic, the cumulative number of cured and discharged cases reached 78,028, and the number of existing suspected cases and new cases significantly decreased, thus suggesting that the spread of the COVID-19 outbreak was controlled in a very effective manner.

Since the pandemic outbreak, the general public has been responsive to the national call to stay at home in quarantine. Yet, given that COVID-19 and pandemic situations are poorly understood, as well as the inconvenience of life and work caused by home quarantine, the public was likely to experience doubts and anxiety related to the disease, while patients with chronic diseases worried about changes of conditions or increased risk of infection when going out for medication.

WeChat is one of the most important Chinese multi-purpose messaging, social media, and mobile payment app, which has over 1 billion users in China [2] and has the advantages of immediacy, interactivity, and convenience. It is also one of the important communication tools for health education in the current “non-contact” service [3, 4]. Therefore, on January 30, 2020, we used WeChat to establish the “COVID-19 Voluntary Assistance Group in Sichuan and Chongqing regions”, dedicated to providing professional consultation and psychological counseling services for Sichuan-Chongqing residents during the COVID-19 outbreak.

In this study, we statistically analyzed consultations initiated by residents in Sichuan and Chongqing regions through WeChat during the COVID-19 outbreak so as to understand the concerns of residents in Sichuan and Chongqing regions, disease development, etc. Furthermore, the advantages and disadvantages of the WeChat consultation methods were analyzed, thus providing a reference for “non-contact” voluntary service platforms implementing consultations.

## Methods

### Data source

The consultation records from January 30, 2020, to March 1, 2020, provided by the WeChat “COVID-19 Voluntary Assistance Group in Sichuan and Chongqing regions” were collected and knowledge on popular science, expert advice, psychological comfort and popular science articles, as well as other information available from the WeChat group, were analyzed. Knowledge on popular science related to the popularization of scientific knowledge associated with COVID-19. Expert advice referred to the consultation answers and corresponding advice related to COVID-19 given by doctors, pharmacists, nurses, and social workers. Psychological comfort included the professional psychological counseling advice offered by psychological consultants for residents quarantined at home during the COVID-19 outbreak. Popular science articles were sorted by collecting knowledge points related to COVID-19.

### Data analysis

Excel was used to summarize and analyze the information, including the composition of experts in the WeChat group, consultation contents, number of consultation items, and answers (reasonable or wrong answers proved by subsequent evidence-based evidence or the official information of the National Health Commission), after which the primary, secondary and general factors of the contents were analyzed by Pareto chart.

### Plotting of Pareto chart

The histogram and broken line graph were plotted with the consultation contents as the abscissa and the number of consultation items as the ordinate, and the two were combined into a Pareto chart. According to the classification principle of Pareto chart, the cumulative constituent ratio of 0–80% was the primary factor (category A); > 80–90% secondary factor (category B); > 90–100% general factor (category C) [5].

## Results

### Composition of assistance experts

This WeChat assistance group had 19 working groups, including 2 social workers and 17 health care workers (5 Western medicine doctors, 1 Traditional Chinese medicine doctor, 4 psychological consultants, 5 nurses, and 2 pharmacists, one of whom was a senior doctor from a Beijing hospital who actively participated in fighting severe acute respiratory syndrome (SARS) in 2003).

### Inclusion criteria for members seeking consultations

Inclusion criteria for members in the WeChat group were following: ordinary home quarantined residents in cities and towns in Sichuan and Chongqing, with different educational level and family income; age: 30–60 years; those with only basic medical knowledge, and those able to use mobile phones to master WeChat operations.

### Consultation content

**Fig. 1:**
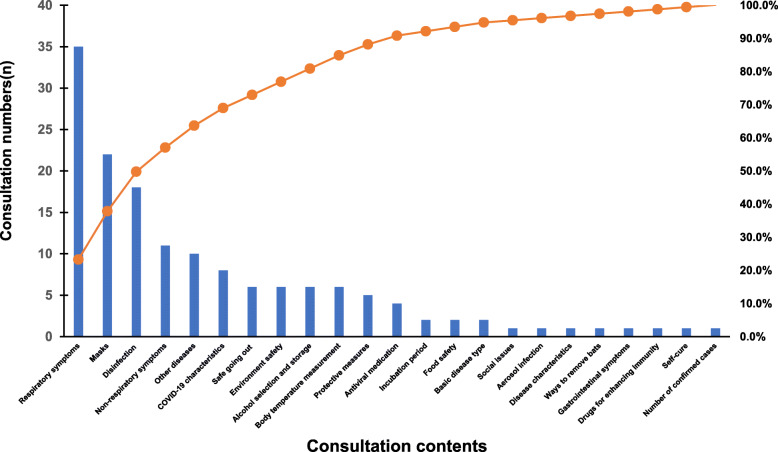
Pareto chart of various consultation contents

The consultation content included 23 categories. The classification and constituent ratio are shown in Table 1, and the Pareto chart of various consultation contents are shown in Fig. 1.

**Table 1:** Classification and constituent ratio of consultation content

No.	Consultation contents	Number of consultation contents (n)	Constituent ratio (%)	Cumulative constituent ratio (%)	Classification of factor^a^
1	Respiratory symptoms	35	23.18	23.28%	A
2	Masks	22	14.57	37.85%	A
3	Disinfection	18	11.92	49.77%	A
4	Non-respiratory symptoms	11	7.28	57.05%	A
5	Other diseases	10	6.62	63.68%	A
6	COVID-19 characteristics	8	5.30	68.98%	A
7	Safe going out	6	3.97	72.95%	A
8	Environment safety	6	3.97	76.92%	A
9	Alcohol selection and storage	6	3.97	80.90%	B
10	Body temperature measurement	6	3.97	84.87%	B
11	Protective measures	5	3.31	88.18%	B
12	Antiviral medication	4	2.65	90.83%	C
13	Incubation period	2	1.32	92.15%	C
14	Food safety	2	1.32	93.48%	C
15	Basic disease type	2	1.32	94.80%	C
16	Social issues	1	0.66	95.47%	C
17	Aerosol infection	1	0.66	96.13%	C
18	Disease characteristics	1	0.66	96.79%	C
19	Ways to remove bats	1	0.66	97.45%	C
20	Gastrointestinal symptoms	1	0.66	98.11%	C
21	Drugs for enhancing immunity	1	0.66	98.78%	C
22	Self-cure	1	0.66	99.44%	C
23	Number of confirmed cases	1	0.66	100.00%	C
Total		151	100		

^a^Category A represents cumulative constituent ratio of 0–80%; Category B cumulative constituent ratio of 80–90%; Category C cumulative constituent ratio of 90–100%

The respiratory symptoms, masks, and disinfection were top three topics identified in the consultation contents, with the respiratory symptoms being predominant (*n* = 35, 23.18%); there were 22 mask related consultations, which accounted for 14.57%, and 18 disinfection related consultations, which accounted for 11.92%; the cumulative constituent ratio of the above three was 49.77%. Besides, consultations related to non-respiratory symptoms, other diseases, COVID-19 characteristics, going out, and environmental safety ranged between 0 and 80%, thus resulting as the primary factors of the consultation contents, i.e., category A. The cumulative constituent ratio of alcohol selection and storage, body temperature measurement and protective measures ranged between 80 and 90%, which suggested these were the secondary factors of the consultation contents, i.e., category B. The remaining 12 types of questions were the general factors of the consultation content, namely category C.

### Changes in the number of consultation items

**Fig. 2:**
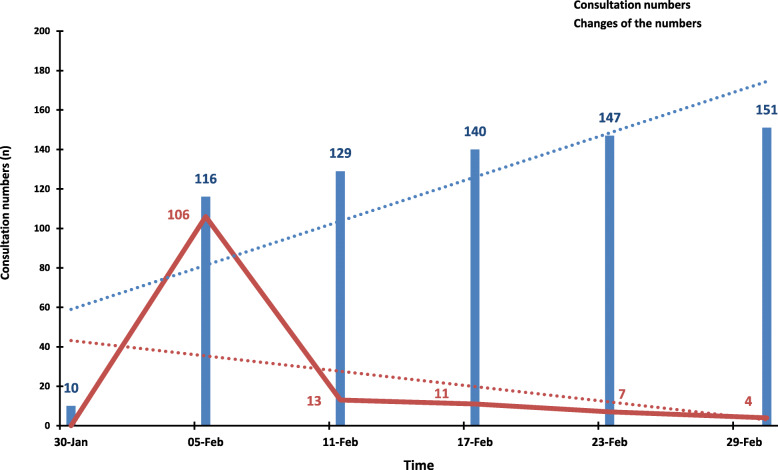
Changing trends in the number of consultation items

From January 30 to March 1, the number of consultation topics showed an increasing trend, which was the most significant in the first week, with 10 consultation topics that were recorded on the first day increasing to 116 on the 7th day. From the second week, the increment leveled off, and the increase rate gradually decreased from 11.2 to 2.7%. Details are shown in Table 2 and Fig. 2.

**Table 2:** Number and changes of consultation contents

Consultation time	Number of consultation contents (n)	Changes of number (n)	Increase rate (%)
January 30	10	0	0.0
February 5	116	106	1060.0
February 11	129	13	11.2
February 17	140	11	8.5
February 23	147	7	5.0
March 1	151	4	2.7

### Answers

There were 307 members in this WeChat group. As of March 1, 151 consultation questions were received; 130 questions (86.1%) related to COVID-19 and 21 (13.9%) unrelated to COVID-19. The specific answers are shown in Table 3.

**Table 3:** Answers to various consultation contents

No.	Consultation content	Number of consultation contents (n)	Answerer	Reasonable answer (n)	Wrong answer (n)	No answer (n)
1	Respiratory symptoms	35	Doctor	33	1	1
2	Masks	22	Doctor	22	0	0
3	Disinfection	18	Doctor	16	2	0
4	COVID-19 characteristics	8	Doctor	6	2	0
5	Safe going out	6	Doctor	6	0	0
6	Environment safety	1	Group friend	1	0	0
		5	Doctor	5	0	0
7	Alcohol selection and storage	1	Group friend	1	0	0
		5	Doctor	3	2	0
8	Body temperature measurement	6	Doctor	6	0	0
9	Protective measures	5	Doctor	5	0	0
10	Antiviral medication	4	Doctor	3	0	1
11	Incubation period	2	Doctor	1	1	0
12	Food safety	2	Doctor	1	1	0
13	Basic disease type	2	Doctor	2	0	0
14	Social issues	1	Doctor	1	0	0
15	Aerosol infection	1	Doctor	1	0	0
16	Disease characteristics	1	Doctor	1	0	0
17	Ways to remove bats	1	Doctor	1	0	0
18	Gastrointestinal symptoms	1	Doctor	0	0	1
19	Drugs for enhancing immunity	1	Doctor	1	0	0
20	Self-cure	1	Doctor	0	1	0
21	Number of confirmed cases	1	Doctor	1	0	0
	Total	130		117	10	3
1	Non-respiratory symptoms	3	Group friend	0	3	0
		8	Doctor	8	0	0
2	Other diseases	10	Doctor	10	0	0
	Total	21		18	3	0
	Total	151		135	13	3

Statistical analyses revealed: answers to 135 questions were confirmed to be reasonable (89.4%) by subsequent evidence-based evidence or official information; answers to 8 questions were erroneous, as was confirmed by the subsequent evidence-based evidence or experts (5.3%). Some of the erroneous answers were following: cooking at high-temperature can be used to disinfect masks, children are not susceptible to the virus, 60 °C alcohol can be used to achieve disinfection, and packaging bags do not need to be disinfected. Answers for the same questions were later corrected by experts; 3 questions were reasonably answered by group friends as was confirmed by experts in the group; 3 questions were left with no answer.

### Summary of other information

A total of 181 other messages related to the pandemic were sent in the WeChat group, including popular science articles, knowledge on popular science, interactive games, and similar. Among them, the number of artciles on popular science was the largest, totaling 84 cases (36.7%), followed by the knowledge on popular science, with a total of 48 cases (21.0%); the expert’s advice ranked third, with 34 cases (14.8%). Details are shown in Table 4.

**Table 4:** Summary of relevant information related to the pandemic

Type	Contents	Number (n)	Wrong contents (n)
Popular science articles	Recommendations of contents related to pandemic online	84	0
Popular science knowledge	COVID-19 and disease knowledge points	17	9
	Mask knowledge points	17	0
	The difference between medical alcohol and industrial alcohol	3	0
	Introduction of other disinfectants	3	0
	Identification of wind-heat and wind-cold cold	2	0
	The difference between disinfection and sterilization	2	0
	Basic diseases	1	0
	COVID-19 symptoms	1	0
	The difference between common cold and viral pneumonia	1	0
	Shuanghuanglian efficacy	1	0
	Total	48	9
Expert advice	Giving residents the right guidance to fight the pandemic	34	0
Psychological counseling	Giving residents sentences of psychological comfort	13	0
Family interactive games	Residents play games with their children at home	2	0
	Total	181	9

## Discussion

This cross-sectional study investigated the use of the WeChat platform by health care workers in providing assistance services for residents in Sichuan and Chongqing during the COVID-19 outbreak. Our results suggested that this model was in line with the current new type of e-Patients who use Internet to interact with doctors and other patients that can solve the disease and life-related problems to a certain extent, and provide the public with psychological support and comfort. Even though China has entered the stable period of COVID-19, countires abroad are still struggling with the rapid outbreak and increase of the disease, while special drugs or vaccines for the treatment of COVID-19 are still lacking. In such context, the implementation of the home quarantine method is highly recommended. Therefore, the “non-contact” e-Patients model similar to the one offered by the WeChat platform may have an important role and a large demand in the future [6].

As shown in the Pareto chart, the three questions related to “respiratory symptoms, masks, and disinfection” were the primary factors in consultation content, accounting for approximately 50% of the total consultation content. This indicated that at the early stage of the outbreak, the public was mainly concerned about how to identify COVID-19 infection symptoms and how to properly implement effective disinfection measures. “Non-respiratory symptoms, other diseases, COVID-19 characteristics, going out, and environmental safety” were also the primary factors identified in the consultation contents. Among these, “non-respiratory symptoms and other diseases” that were not related to COVID-19 ranked the top, and “other diseases” mainly referred to follow-up management of chronic diseases. The two consultation questions were related to the home quarantine of patients with chronic diseases not able to go out to visit the doctor at any time.

Over recent years, multiple studies have shown that WeChat can be used to deliver relevant assistance for effective remote management of chronic diseases, thus providing great convenience to patients [7, 8]. Therefore, under special circumstances such as pandemic situations, the “non-contact” consultation similar to the one offered through WeChat can be easily used by chronic disease patients and effectively solve the problem of seeking medical treatment.

“Alcohol selection and storage, body temperature measurement, protective measures” were the secondary factors identified in the consultation content. These questions suggested that the public was not very clear about disinfection, body temperature, and basic protection, etc., and professional answers and help were urgently needed at the critical stage of the pandemic. Also, “therapeutic drugs, incubation period, disease characteristics, self-cure conditions, and the number of confirmed cases” were the general factors in the consultation content. These results suggested that since the general public did not have a good understanding of the previously unknown COVID-19, the scope of the consultation content was wide, and some consultation questions were beyond the scope of medical care. This further suggested that similar volunteer service platforms in the late stage of the pandemic may also benefit from the assistance from multidisciplinary professionals [9, 10], such as experts in environmental protection, statistics, zoology, etc., so as to deliver more comprehensive and informed consultation services to the public.

Since the establishment of this WeChat group on January 30, the number of consultation items has generally shown an increasing trend, especially in the first week when the number of consulted items significantly increased from 10 on the first day to 116 in the first week. The number of consultation items has significantly increased over a short period, which may be related to the attitude toward the public’s unknown and panic psychology at the early stage of the outbreak [11], which needed to be addressed by medical experts providing professional answers and psychological counseling. Multiple studies [12–15] have revealed that WeChat consultation services can deliver effective, convenient, and timely help to the general public.

Since the second week, the increase in the consultation content leveled off, and the increase rate gradually decreased from 11.2 to 2.7%. This can be explained by the fact that the news actively addressed the spread of the pandemic, thus disseminating the relevant knowledge, after which strong emergency control measures were adopted across the country, so the spread of the pandemic began to stabilize, and the public had positive perception and self-efficacy to overcome the pandemic [16]. In the face of various public health emergencies posing a great threat to human life such as the SARS in 2003, the Wenchuan earthquake, and COVID-19, the public is prone to anxious and nervous psychological reactions [17, 18], so psychological interventions after public health emergencies are as important as effective disaster control. This suggests that in the late stage of this pandemic, psychologists may need to provide long-term psychological treatment and help the general public, especially those in Wuhan.

As of March 1, 151 consultation questions were received, among which 130 (86.1%) were related to COVID-19. These were also predominant items among the consultation content, which was consistent with the patient’s concern during the pandemic. There were eight wrong answers to consultation questions related to the spread of the pandemic. For example, at the early stage of the pandemic, it was thought that “children were not susceptible”. However, the evidence-based practice showed that age was not a relevant factor in resistance to the COVID-19 and that all people were susceptible under appropriate conditions. The National Health Committee clearly stated on February 2 that “Children and pregnant women are susceptible to COVID-19” [19, 20]. Another example relates to “60 °C alcohol can achieve disinfection”, which was later on disapproved by the guidelines recommending that only 75% of ethanol had a disinfection effect [21]. These erroneous answers suggest that data should be collected and shared in a timely manner as the pandemic spreads so as to help update information on the pandemic and accurately control it.

Besides, two other questions were, “Is the simple gastrointestinal disturbance symptom of the COVID-19 infection?” and “Can drugs that are used to treat the acquired immunodeficiency syndrome (AIDS) and flu in Thailand treat COVID-19?” were asked at the early stage of the outbreak. Still, because at that time, the gastrointestinal symptoms caused by COVID-19 and the medication regimen for COVID-19 in Thailand were unclear, doctors could not give a specific answer. This suggested that during the spread of the pandemic, unclear situations may occur. Health care workers cannot give answers that are not supported by evidence, and it is necessary to pay attention to the current evidence-based practice before giving answers.

Among other materials in the WeChat group, the “popular science articles” on the Internet accounted for the largest proportion. After selection, experts sent them to the group for public learning. In the summary of knowledge on popular science, “COVID-19 and disease info” and “mask info” focused on the public concern. Since experts focused on these two aspects of knowledge popularization, their proportion was the highest. At the same time, during the consultation process, it was found that a few residents had psychological problems, such as panic, nervousness, anxiety, etc. Since these negative emotions may also be spread through social networks [22], positive psychological counseling could alleviate public emotions. This suggests that in the face of the pandemic situation, the information should be summarized in a timely manner, and attention should be paid to public needs.

This study has some limitations. First, the general data on the members’ age, gender distribution, academic background, family income, residence distribution, etc., were not complete because of the urgent establishment of the WeChat group, which may have partialinfluence on the analysis results. Second,COVID-19 was more likely to occur in young and middle-aged people, according to the early real-time data released by the National Health Commission of the People’s Republic of China. The young and middle age of onset has been confirmed in the following research reports, for instance, the average age was 55.5 [23], 59 [24] or 46 [25] years and over 70% of the patients were 30–69 years old [25]. However, only people between 30 and 60 were included in our study,which made it impossible to determine whether the problems encountered in this study were public concerns. Third, the included assistants had limited professional field, which led to failure to answer public questions in a timely manner, wrong answers, and delayed update of pandemic information, etc. Therefore, it is necessary to establish a multi-disciplinary expert team to continuously collect the latest pandemic information and share data, thereby providing better consultation services for residents staying at home. Fourth, this study only collected the WeChat group consultations from home quarantine patients in Sichuan and Chongqing, while relevant data from other domestic areas (such as Beijing, Shanghai, Guangzhou, etc.) or those from other social platforms (such as Weibo, QQ, Tik Tok, Kuaishou, Douban, Zhihu, Tieba, etc.) were not collected that led to limited data and information, which may not fully reflect the public concerns and disease changes during the pandemic. Finally, the data collection time and sample size in this study were limited.

## Conclusion

This study showed that WeChat can be effectively used for providing professional consultation, psychological support, and other assistance services to the public during the pandemic, and thus should be promoted. The relevant work experience can be used as a reference for subsequent similar “non-contact” e-Patients services, and the service model can be optimized by summarizing consultation questions and organizing them into manuals or self-service answers, so as to better serve the home quarantine residents.

## Data Availability

All data generated or analysed during this study are included in this published article.

## Abbreviations

COVID-19: Coronavirus Disease 2019
SARS: Severe acute respiratory syndrome
AIDS: Acquired immunodeficiency syndrome
